# Key roles in copper efflux and protein homeostasis of the intrinsically disordered region of a bacterial outer membrane channel

**DOI:** 10.1016/j.jbc.2025.110670

**Published:** 2025-09-01

**Authors:** Amira Khochtali, Marine Ote, Hugo Bâlon, Marc Dieu, Patricia Renard, Catherine Michaux, Jean-Yves Matroule

**Affiliations:** 1 Research Unit in Biology of Microorganisms (URBM), Department of Biology, Namur Research Institute for Life Sciences (NARILIS), University of Namur, Namur, Belgium; 2 Laboratoire de Chimie Physique des Biomolécules, Department of Chemistry, University of Namur, Namur, Belgium; 3 Namur Research Institute for Life Sciences (NARILIS), Namur Institute of Structured Matter (NISM), Namur, Belgium; 4 MaSUN, Mass Spectrometry Facility, University of Namur, Namur, Belgium

**Keywords:** bacteria, copper, efflux, intrinsically disordered region, stress response, *Caulobacter*, PcoA, PcoB

## Abstract

Metals like copper (Cu), zinc, and nickel exhibit dual nature, necessitating a tight regulation of their cellular homeostasis to meet physiological demands while preventing toxicity. In bacteria, metal homeostasis involves inner membrane P-type ATPases and ABC transporters, envelope-spanning tripartite efflux pumps, and outer membrane pore-forming proteins. Four decades ago, the outer membrane β-barrel protein PcoB was shown to provide an additional layer of Cu resistance in an *Escherichia coli* strain isolated from the gut of swine fed with Cu supplements. Interestingly, most PcoB homologs contain a poorly conserved disordered N-terminal domain (NTD) rich in histidine and methionine residues, which are commonly associated with Cu coordination in cuproproteins. This suggests a potential role for the NTD in PcoB-mediated Cu efflux. We previously demonstrated that the free-living bacterium *Caulobacter vibrioides* primarily relies on PcoB for Cu homeostasis. Here, we show that the NTD of *C. vibrioides* PcoB is critical for PcoB function and stability, tolerating the swapping with the poorly conserved *E. coli* PcoB NTD and significant truncations. Unexpectedly, the predicted signal peptide was dispensable, challenging traditional concepts of protein translocation mechanisms. Moreover, the PcoB NTD plays a surprising role in stabilizing the periplasmic multicopper oxidase PcoA, encoded within the same operon as PcoB, highlighting a new role for an intrinsically disordered region.

Since the 19th century, copper (Cu) has been widely recognized as an effective antimicrobial agent because of its toxicity at high concentration, which mainly results from mismetallation events (1), reactive oxygen species production (2, 3) and protein aggregation (4).

Bacteria often encounter Cu in their natural environments, where it can be found in sediments or accumulate in mammalian macrophages as a defense mechanism against bacterial infections (5, 6, 7). Consequently, bacteria have evolved Cu resistance strategies to survive these harsh conditions (8). The most conserved Cu resistance strategies include (a) intracellular or extracellular Cu ion chelation by metallothioneins, bufferins, and glutathione for instance (9, 10), (b) oxidation of Cu(I) ions to the less toxic Cu(II) ions by multicopper oxidases such as CueO (11), and (c) Cu efflux. In Gram-negative bacteria, Cu efflux is achieved through the combined action of (i) inner membrane (IM) P1B-type ATPases that transport Cu(I) ions from the cytoplasm to the periplasm (12) and (ii) tripartite HME–RND pumps such as CusABC spanning the bacterial envelope and use the proton motive force (PMF) to expel periplasmic and potentially cytoplasmic Cu(I) out of the cell with the help of a periplasmic chaperone such as CusF (13).

In the early 1980s, an additional seven-component Cu resistance system was identified in an *Escherichia coli* strain isolated from the feces of swine fed with a Cu-rich diet (14, 15). This system, known as the Pco system, is encoded by a plasmid-borne operon and has been poorly characterized so far. Among its components, only the multicopper oxidase PcoA has been identified as a close homolog of the CueO multicopper oxidase (16). The other Pco components are believed to be involved in periplasmic Cu chelation and efflux into the extracellular medium (16). In *Acinetobacter baumannii*, PcoA and PcoB mutants display a higher Cu sensitivity and an attenuated phenotype in a *Galleria mellonella* infection model (17).

Recently, we have shown that the aquatic α-proteobacterium *Caulobacter vibrioides* harbors a chromosomal two-gene operon encoding the PcoA multicopper oxidase and the outer membrane (OM) β-barrel protein PcoB, both involved in Cu resistance (18). PcoA and PcoB are expressed in sessile stalked cells and facilitate rapid Cu detoxification and efflux, favoring DNA replication and cell division (18).

Recently, we have experimentally and bioinformatically shown that PcoB preferentially binds Cu(II) ions and assembles into a β-barrel harboring a disordered N-terminal tail (19).

Given the OM location of PcoB, the mechanism underlying PcoB-mediated Cu efflux remains unclear and very likely differs from the well-known metal efflux ATPases and HME–RNDs that are powered by ATP hydrolysis and the PMF, respectively.

In the present study, we investigated the role of the disordered N-terminal region of PcoB in Cu resistance. We provided evidence that this region contributes to PcoB's function and stability and more surprisingly to PcoA stability, highlighting a new role for an intrinsically disordered region (IDR). In addition, PcoB seems to harbor an atypical signal peptide (SP), challenging existing paradigms on protein translocation across the IM.

## Results

### The disordered PcoB N-terminal domain is required for Cu resistance

**Figure 1:**
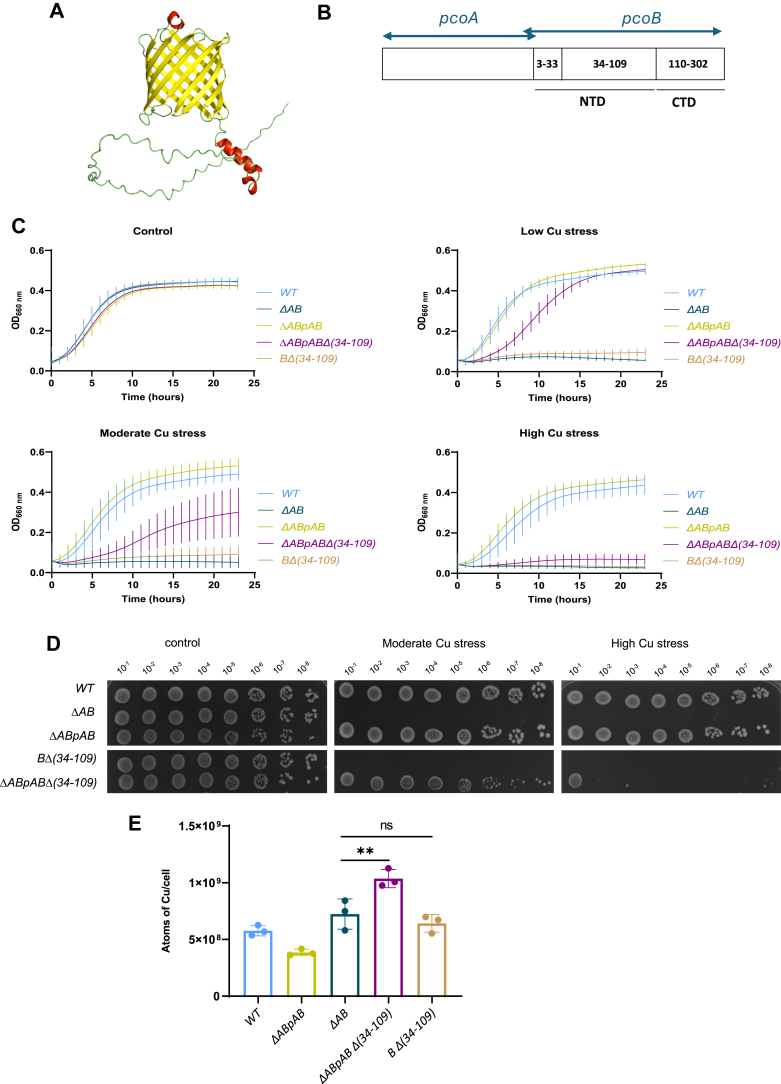
**The disordered PcoB N-terminal domain is required for copper (Cu) resistance.***A,* structure prediction of PcoB using AlphaFold3. *B,* organization of *pcoAB* operon. *C,* growth profiles at an absorbance of 660 nm of WT, *ΔAB*, *ΔABpAB*, *ΔABpABΔ(34–109)*, and *BΔ(34–109)* strains grown in PYE medium (*top left*) and in PYE medium supplemented with CuSO_4_ (*top right* and *down*). Mean ± SD, at least three biological replicates. *D,* viability assay on PYE plates of WT, *ΔAB*, *ΔABpAB*, *BΔ(34–109)*, and *ΔABpABΔ(34–109)* strains, in control, moderate, and high CuSO_4_ stress condition. *E,* number of Cu atoms per cell exposed to 175 μM CuSO_4_ for 5 min. Individual values, mean ± SD, and at least three biological replicates are represented. *p* Values were calculated using ANOVA combined with Dunnett's multiple comparison test (∗*p* < 0.05, ∗∗*p* < 0.01, ∗∗∗*p* < 0.001, and ∗∗∗∗*p* < 0.0001) (Table S1). PYE, peptone yeast extract.

The predicted 3D structure of PcoB by AlphaFold3 is consistent with its OM location and reveals two key features: (1) a C-terminal domain (CTD) organized into β sheets with 12 antiparallel β-strands forming a small β-barrel and (2) a 109-residue-long IDR as the N-terminal domain (NTD) with one short α-helix encompassing residues 91 to 104 (Fig. 1*A*). This predicted structure aligns with the crystal structures of the PcoB β-barrel from *E. coli* and with the preliminary diffraction data from *A. baumannii*, which were resolved at a 2.0 Å and 6.5 Å resolution, respectively (20, 21). However, the high flexibility of the NTD prevented the crystallization of the full protein, which was therefore limited to the β-barrel.

The β-barrel regions of PcoB (residues 110–302) and 7PGE superimpose with an RMSD of 0.632 Å over 131 Cα atoms, indicating high structural similarity (Fig. S1).

In *C. vibrioides*, the PcoB intrinsically disordered NTD represents more than 30% of total protein sequence, classifying PcoB as a hybrid intrinsically disordered protein (IDP) (19). PcoB NTD also contains 17 histidine (His) and 5 methionine (Met) residues and is very likely oriented toward the periplasm (19). His and Met residues are frequently involved in Cu coordination (22), suggesting that the PcoB NTD may bind Cu and facilitate Cu efflux.

As an OM protein, PcoB is supposed to harbor an SP for translocation through the IM *via* the Sec translocon. Surprisingly, no SP could be detected in PcoB using the SP prediction tool SignalP-5.0 (Fig. S2*A*). Despite the extensive diversity of bacterial Sec SPs, certain features are conserved in all Sec substrates, including a three-region design: a positively charged N-terminal region (n-region), a hydrophobic central region (h-region), and a neutral, polar C-terminal region (c-region), along with a three-residue motif for signal peptidase I, typically comprising AXA motifs at the end of the c-region (23). Manual analysis of the PcoB sequence revealed a potential poorly conserved Sec motif, characterized by a high occurrence of hydrophobic residues and AXA patterns at positions 13 to 15, 20 to 22, and 34 to 36 (Fig. S2*B*). To confirm this prediction, we isolated a periplasmic/OM fraction from exponentially growing *C. vibrioides* cells expected to contain the mature PcoB and subjected this fraction to trypsin digestion followed by LC–MS analysis. No peptide corresponding to the first 33 residues of PcoB was detected by LC–MS, suggesting that this peptide was cleaved during PcoB translocation through the IM *via* the Sec translocon (Fig. S2*C*). Therefore, we decided to include the first 33 residues of PcoB as a putative SP in the subsequent genetic constructs to allow correct PcoB addressing to the OM.

To investigate the role of the PcoB NTD_34–109_ in Cu resistance, we deleted the region encompassing the alanine 34 to threonine 109 residues within the chromosomal *pcoB* allele (*BΔ(34–109)*) or within the extrachromosomal *pcoAB* operon cloned in a low-copy pMR10 plasmid under the control of the strong and constitutive pLac promoter (*ΔABpABΔ(34–109)*) (Fig. 1*B*). The growth of the resulting mutant strains was monitored over 24 h in liquid rich peptone yeast extract (PYE) medium under low (50 μM), moderate (100 μM CuSO_4_), and high (150 μM CuSO_4_) Cu stress conditions. The *ΔABpABΔ(34–109)* mutant exhibited an increased Cu sensitivity under moderate Cu stress relative to the WT and the complemented *ΔABpAB* genetic backgrounds (Fig. 1*C*). This Cu sensitivity was concentration dependent, with only a minor growth delay under low Cu stress and complete growth inhibition under high Cu stress (Fig. 1*C*).

The increased Cu sensitivity of the *ΔABpABΔ(34–109)* mutant was also observed on plate, where diluted cultures were spotted on solid PYE medium under moderate (75 μM CuSO_4_) and high (100 μM CuSO_4_) Cu stress (Fig. 1*D*). The *BΔ(34–109)* chromosomal mutant displayed a ΔAB mutant phenotype even under low Cu stress, suggesting a higher impact of the *BΔ(34–109)* allele when present as a single chromosomal copy (Fig. 1, *C* and *D*).

To determine whether the increased Cu sensitivity of the *BΔ(34–109)* and *ΔABpABΔ(34–109)* mutants results from a defect in Cu efflux, we measured the Cu content in total cell extracts using inductively coupled plasma optical emission spectroscopy (ICP-OES). Consistent with the observed Cu sensitivity, the *BΔ(34–109)* and the *ΔABpABΔ(34–109)* mutants displayed no significant difference or a slight significant increase of the intracellular Cu levels relative to the *ΔAB* mutant, respectively (Fig. 1*E*), indicating that the PcoB NTD_34–109_ is crucial for Cu efflux.

### PcoB and PcoA stability relies on the PcoB NTD

**Figure 2:**
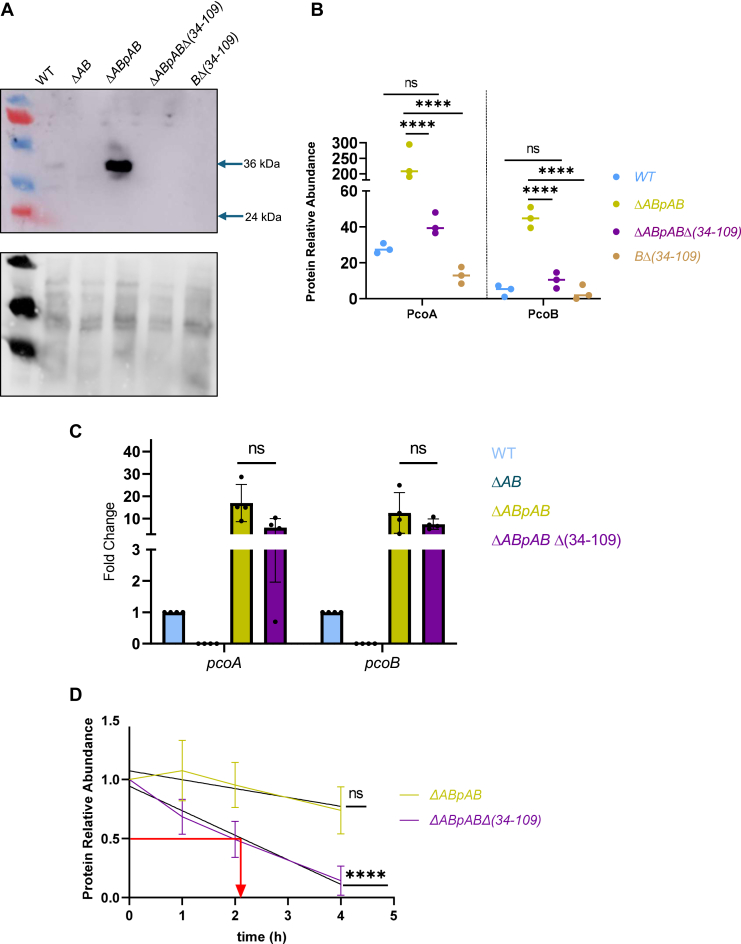
**PcoB and PcoA stability relies on the PcoB N-terminal domain (NTD).***A,* immunoblot anti-PcoB performed on total cell extracts from WT, *ΔAB*, *ΔABpAB, ΔABpABΔ(34–109)*, and *BΔ(34–109)* strains grown in PYE medium. The *lower panel* corresponds to the Cy5-stained gel used as a loading control. *B,* normalized spectrum counts of PcoA and PcoB peptides in WT*, ΔABpAB, ΔABpABΔ(34–109)*, and *BΔ(34–109)* strains grown in PYE medium, measured by LC–MS. Individual values and means represented. *C,* relative *pcoA* and *pcoB* mRNA levels measured by RT–quantitative PCR in the WT, *ΔAB*, *ΔABpAB*, and *ΔABpABΔ(34–109)* strains grown in PYE medium. Individual values, mean ± SD, and at least three biological replicates are represented. *p* Values were calculated using an unpaired *t* test; *∗p <* 0.05 (Table S1). *D,* PcoA abundance measured by LC–MS in total cell extracts obtained 0 h, 1 h, 2 h, and 4 h after chloramphenicol treatment of the *ΔABpAB and ΔABpABΔ(34–109)* strains grown in PYE medium. *p* Values were calculated using a linear regression statistical test; ∗∗∗∗*p* < 0.0001 (Table S1). PYE, peptone yeast extract.

The increased sensitivity of the *BΔ(34–109)* and *ΔABpABΔ(34–109)* mutants could result from a loss of function and/or reduced stability of the mutated PcoB. Consistent with this latter hypothesis, PcoB remained undetectable when performing an anti-PcoB immunoblot on total cell extracts from the *BΔ(34–109)* and the *ΔABpABΔ(34–109*) mutants (Fig. 2*A*).

To rule out any epitope loss in the PcoBΔ(34–109) mutant that could impair anti-PcoB antibody recognition and immunoblot reliability, we quantified PcoB in periplasmic/OM fractions using LC–MS. Consistent with the immunoblot data, the deletion of the PcoB NTD (34–109) domain leads to a 4.4-fold decrease of PcoB relative to the *ΔABpAB* strain used as a control (Fig. 2*B*). Yet, PcoB abundance remained at a WT level in the *ΔABpABΔ(34–109)* mutant. Considering the low expression of the chromosomal *pcoB*, we decided to mainly focus on the plasmidic version of PcoB, where *pcoB* was kept in operon with *pcoA* to ensure the coupled translation of PcoA and PcoB. Indeed, in the *ΔABpB* strain where the sole *pcoB* gene is present on pMR10, PcoB abundance is lower than in the *ΔABpAB* strain (Fig. S4).

Unexpectedly, the LC–MS analysis also revealed a significant 18-fold and 5.6-fold decrease in PcoA levels in *BΔ(34–109)* and in*ΔABpABΔ(34–109)*, respectively (Fig. 2*B*). Accordingly, PcoA abundance is also reduced in the *ΔABpA* background (relative to the *ΔABpAB* background), which mimics a *ΔB* mutant (Fig. S4). This latter observation suggests that PcoB, and more specifically the PcoB NTD_34–109,_ is required to sustain PcoA homeostasis.

It is unlikely that the decrease in PcoA and PcoB abundance observed in the *ΔABpABΔ(34–109)* mutant results from a downregulation of the *pcoAB* operon transcription, considering that the *pcoAB* operon is under the control of the constitutive lac promoter when expressed from the pMR10 plasmid. Consistent with this assumption, the relative amounts of *pcoA* and *pcoB* transcripts measured by RT–quantitative PCR did not significantly vary between the *ΔABpABΔ(34–109)* mutant and the *ΔABpAB* strain (Fig. 2*C*).

To test whether the PcoB NTD_34–109_ could play a role in PcoA and PcoB stability, we aimed to compare their respective half-life in the *ΔABpABΔ(34–109)* and *ΔABpAB* backgrounds. PcoA and PcoB levels were measured by LC–MS in total protein extracts at different time points after translation inhibition with 100 μg/ml chloramphenicol. In the *ΔABpAB* strain, PcoA is relatively stable, with protein levels remaining constant over 4 h after translation inhibition (Fig. 2*D*). However, in the *ΔABpABΔ(34–109)* mutant, PcoA content rapidly decreases after protein translation arrest to a level of 50% after 2 h, indicating that the PcoB NTD_34–109_ contributes to PcoA stability (Fig. 2*D*).

Interestingly, PcoB stability was hardly affected by the deletion of the PcoB NTD_34–109_, at least during the first 4 h of protein translation arrest, suggesting that the lower abundance of the PcoBΔ(34–109) does not result from a post-translational destabilization as it is observed for PcoA (Fig. S5).

Considering that PcoA and PcoB protein levels in the *ΔABpABΔ(34–109)* mutant remain equivalent to the levels measured in the WT strain (Fig. 2*B*), we propose that the Cu sensitivity of the *ΔABpABΔ(34–109)* mutant is, at least partially, because of a complete or partial loss of PcoB function.

### PcoB NTD (34–109) domain is tolerant to sequence and size change

**Figure 3:**
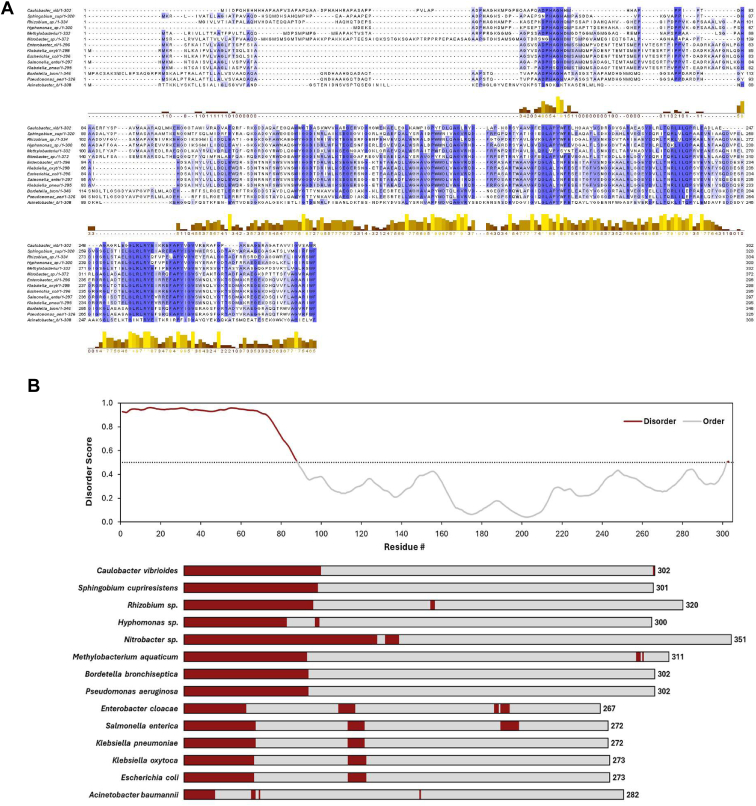
**PcoB N-terminal domain (NTD) is poorly conserved.***A,* multiple MAFFT alignment of PcoB_Cc_ with 13 PcoB homologs from α-, β-, δ-, and γ-proteobacteria. Identical residues are highlighted in *blue*. Predicted conservation and intrinsic disorder profile of PcoB homologs. *B,* RIDAO mean disorder profile (MDP) of *Caulobacter vibrioides* PcoB. Every residue displaying a score above the 0.5 threshold is considered disordered. *C,* schematic representation of MDP derived from RIDAO for 13 homologs of *C. vibrioides* PcoB identified by sequence alignment and conservation. Disordered and ordered regions are highlighted in *dark red* and *grey*, respectively. RIDAO, Rapid Intrinsic Disorder Analysis Online.

The alignment of the *C. vibrioides* PcoB protein sequence with PcoB orthologs from α-, β-, and γ-proteobacteria reveals a poor conservation of PcoB NTD compared with the well-conserved CTD forming the β-barrel (Fig. 3*A*). However, the disorder of PcoB NTD seems to be present in all PcoB orthologs (Fig. 3, *B* and *C*), suggesting that the role of the PcoB NTD may not be directly related to its specific amino acid composition but rather to its length and/or disordered structure.

**Figure 4:**
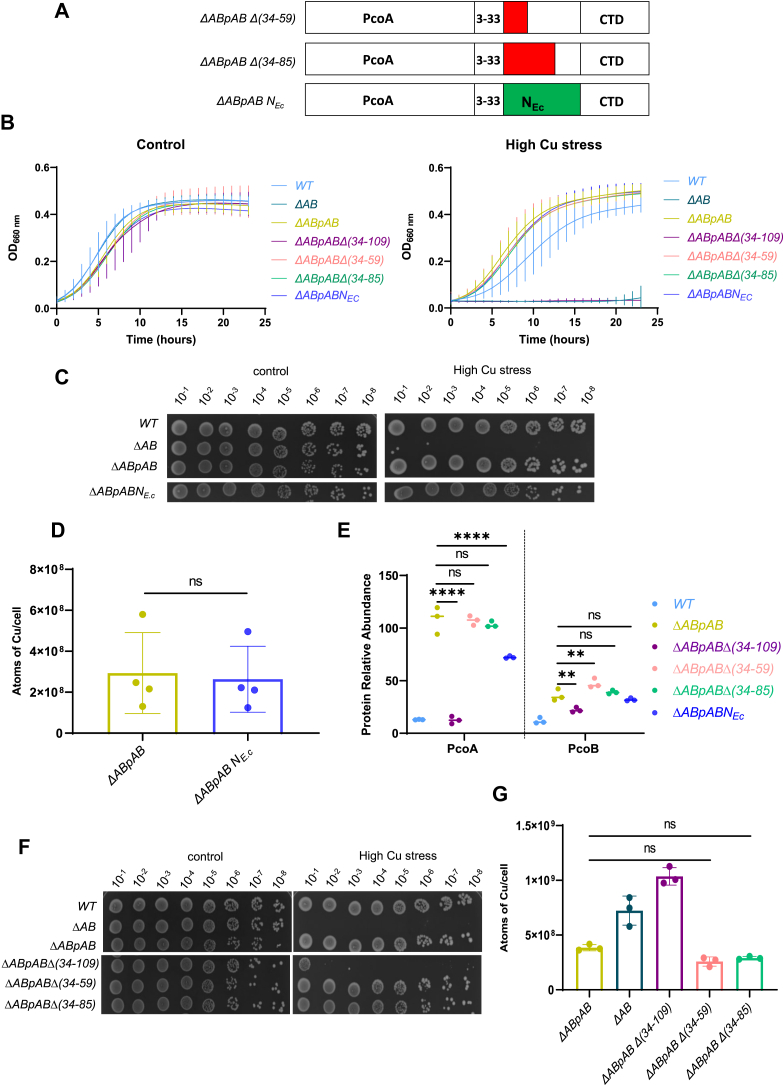
**PcoB N-terminal domain (NTD) is tolerant to sequence and size change.***A,* schematic representation of the *ΔABpABΔ(34–59)*, *ΔABpABΔ(34–85)*, and *ΔABpABN*_*Ec*_ mutants highlighting the deleted regions (in *red*) or the swapped region (*green*). *B,* growth profiles at an absorbance of 660 nm of WT, *ΔAB*, *ΔABpAB*, *ΔABpABΔ(34–109)*, *ΔABpABΔ(34–59)*, *ΔABpABΔ(34–85)*, and *ΔABpABN*_*Ec*_ strains grown in PYE medium (*left*) and in PYE medium supplemented with CuSO_4_ (*right*). Mean ± SD, at least three biological replicates. *C,* viability assay on PYE plates of WT, *ΔAB*, *ΔABpAB*, and *ΔABpABN*_*Ec*_ strains, in control and high CuSO_4_ stress conditions. *D,* number of Cu atoms per cell in the *ΔABpAB* and *ΔABpABN*_*Ec*_ strains exposed to 175 μM CuSO_4_ for 5 min. Individual values, mean ± SD, and at least three biological replicates are represented. *p* Values were calculated using an unpaired *t* test; ∗*p* < 0.05 (Table S1). *E,* normalized spectrum counts of PcoA and PcoB peptides in the WT*, ΔABpAB, ΔABpABΔ(34–109), ΔABpABΔ(34–59), ΔABpABΔ(34–85),* and *ΔABpABN*_*Ec*_ strains grown in PYE medium, measured by LC–MS. Individual values and means represented. *p* Values were calculated using ANOVA combined with Dunnett's multiple comparison test (∗*p* < 0.05, ∗∗*p* < 0.01, ∗∗∗*p* < 0.001, and ∗∗∗∗*p* < 0.0001). *F,* viability assay on PYE plates of WT, *ΔAB*, *ΔABpAB*, *ΔABpABΔ(34–109)*, *ΔABpABΔ(34–59)*, and *ΔABpABΔ(34–85)* strains, in control and high CuSO_4_ stress conditions. The WT*, ΔAB*, and *ΔABpAB* have been reused from Figure 1*D* because all the strains from Figure 4*F* originate from the same plate as the strains from Figure 1*D*. *G,* number of Cu atoms per cell in the *ΔABpAB, ΔAB, ΔABpABΔ(34–109), ΔABpABΔ(34–59)*, and *ΔABpABΔ(34–85)* strains exposed to 175 μM CuSO_4_ for 5 min. Individual values, mean ± SD, and at least three biological replicates are represented. Values for the *ΔABpAB, ΔAB*, and *ΔABpABΔ(34–109)* are the same as in Fig. 1E as it is a unique dataset. *p* Values were calculated using ANOVA combined with Dunnett's multiple comparison test (∗*p* < 0.05, ∗∗*p* < 0.01, ∗∗∗*p* < 0.001, and ∗∗∗∗*p* < 0.0001) (Table S1). PYE, peptone yeast extract.

To test this hypothesis, we swapped the NTD_34–109_ of *C. vibrioides* PcoB with the NTD_25–90_ of *E. coli* PcoB (*ΔABpABN*_*Ec*_), keeping the first 33 residues from *C. vibrioides* PcoB as an SP (Fig. 4*A*), and tested the ability of the resulting chimeric PcoB to sustain Cu resistance. The *ΔABpABN*_*Ec*_ strain shows similar growth pattern to the *ΔABpAB* strain under control conditions and under any of the tested Cu stress conditions in liquid medium (Fig. 4*B*) or on plates (Fig. *C*). Consistent with this observation, the *ΔABpABN*_*Ec*_ mutant displayed an intracellular Cu content equivalent to that of the *ΔABpAB* strain (Fig. 4*D*). Accordingly, the PcoB protein levels were not affected by the domain swapping, whereas PcoA levels were significantly reduced, yet remaining much higher than in the *ΔABpABΔ(34–109)* mutant (Fig. 4*E*).

Together, these results indicate that the overall primary sequence of the PcoB NTD_34–109_ does not seem to be essential for PcoB stability and function.

Considering the apparent tolerance of the PcoB NTD_34–109_ to sequence change, we questioned whether the length of this disordered NTD might be a limiting factor. To address this point, we generated the *ΔABpABΔ(34–59)* and *ΔABpABΔ(34–85)* mutants, missing the first 26 and 52 residues, respectively (Fig. 4*A*).

Surprisingly, these mutants phenocopied the *ΔABpA*B strain in liquid medium (Fig. 4*B*) and on plates (Fig. 4*F*) under any of the tested Cu stress conditions. Consistent with these observations, the intracellular Cu content (Fig. 4*G*) and the PcoA level (Fig. 4*E*) of these mutants was comparable to the *ΔABpAB* strain under Cu stress. The PcoB level was slightly higher in *ΔABpABΔ(34–59)* than in ΔABpAB. These results indicate that the PcoB NTD_34–109_ can be significantly shortened without affecting the stability of PcoB and PcoA or the function of PcoB.

### Cu resistance relies on a minimal set of His residues within PcoB NTD

**Figure 5:**
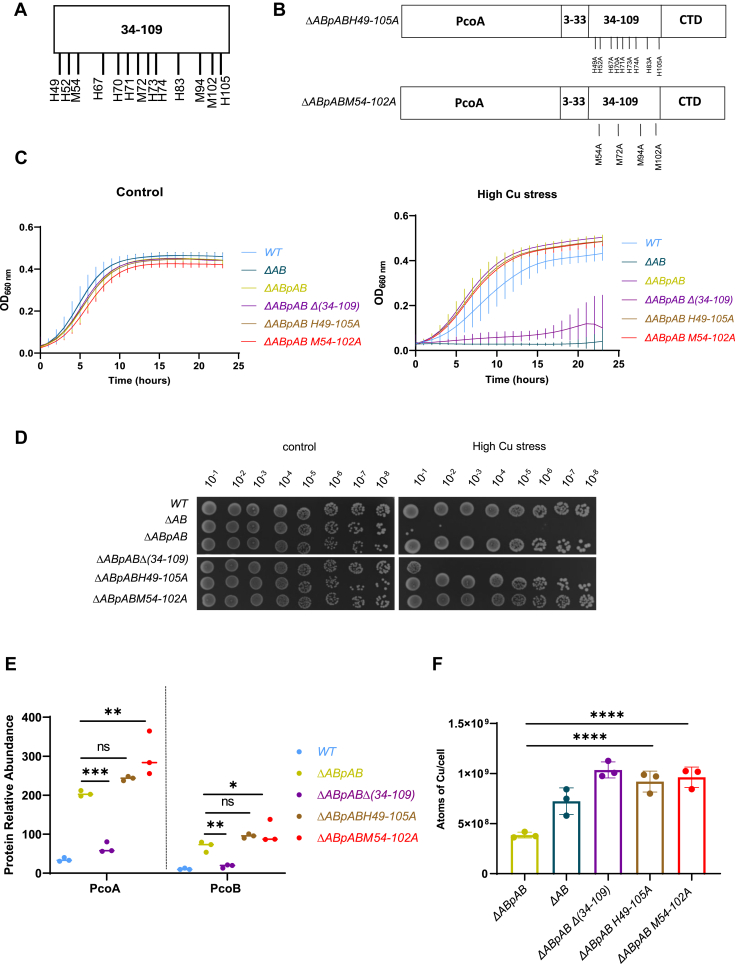
**Methionine (Met) and histidine (His) residues of PcoB N-terminal domain (NTD) play a key role in PcoB function.***A,* schematic representation of the His and Met residues located in the PcoB 34–109 region. *B,* schematic representation of the *ΔABpABH49–105A* and *ΔABpAM54–102A* mutants. *C,* growth profiles at an absorbance of 660 nm of WT, *ΔAB*, *ΔABpAB*, *ΔABpABΔ(34–109)*, *ΔABpABH49–105A*, and *ΔABpABM54–102A* strains grown in PYE medium (*left*) and in PYE medium supplemented with CuSO_4_ (*right*). *D,* viability assay on PYE plates of WT, *ΔAB*, *ΔABpAB*, *ΔABpABΔ(34–109)*, *ΔABpABH49–105A*, and *ΔABpABM54–102A* strains, in control and high CuSO_4_ stress conditions. *E,* normalized spectrum counts of PcoA and PcoB peptides in the WT*, ΔABpAB, ΔABpABΔ(34–109), ΔABpABH49–105A*, and *ΔABpABM54–102A* strains grown in PYE medium, measured by LC–MS. Individual values and means represented. *p* Values were calculated using ANOVA combined with Dunnett's multiple comparison test (∗*p* < 0.05, ∗∗*p* < 0.01, ∗∗∗*p* < 0.001, and ∗∗∗∗*p* < 0.0001). *F,* number of Cu atoms per cell in the *ΔABpAB, ΔAB, ΔABpABΔ(34–109)*, *ΔABpABH49–105A,* and *ΔABpABM54–102A* strains exposed to 175 μM CuSO_4_ for 5 min. Individual values, mean ± SD, and at least three biological replicates are represented. Values for the *ΔABpAB, ΔAB,* and *ΔABpABΔ(34–109)* are the same as in Figure 1*E* as it is a unique dataset. *p* Values were calculated using ANOVA combined with Dunnett's multiple comparison test (∗*p* < 0.05, ∗∗*p* < 0.01, ∗∗∗*p* < 0.001, and ∗∗∗∗*p* < 0.0001) (Table S1).

The presence of nine His and four Met residues in the PcoB NTD_34–109_ (Fig. 5*A*) led us to hypothesize that these residues may bind Cu ions and play a key role in PcoB function. Therefore, we mutated these His (H) or Met (M) residues into Ala (A), yielding the *ΔABpABH49–105A* (H49, H52, H67, H70, H71, H73, H74, H83, and H105) and the *ΔABpABM54–102A* (M54, M72, M94, and M102) mutants (Fig. 5*B*).

These multiple point mutants exhibited a similar growth profile to the *ΔABpAB* strain under both control and Cu stress conditions (Fig. 5*C*), which was further confirmed on plate (Fig. 5*D*). Consistent with these observations, the PcoA and PcoB levels were not negatively affected by the point mutations (Fig. 5*E*). Surprisingly, mutating the His and Met residues in the PcoB NTD_34–109_ led to a striking Cu accumulation, suggesting that these residues play a key role in PcoB-dependent Cu efflux (Fig. 5*F*). The lack of Cu sensitivity of these multiple point mutants might therefore be due to the high level of PcoA detoxifying the high amount of accumulated Cu.

**Figure 6:**
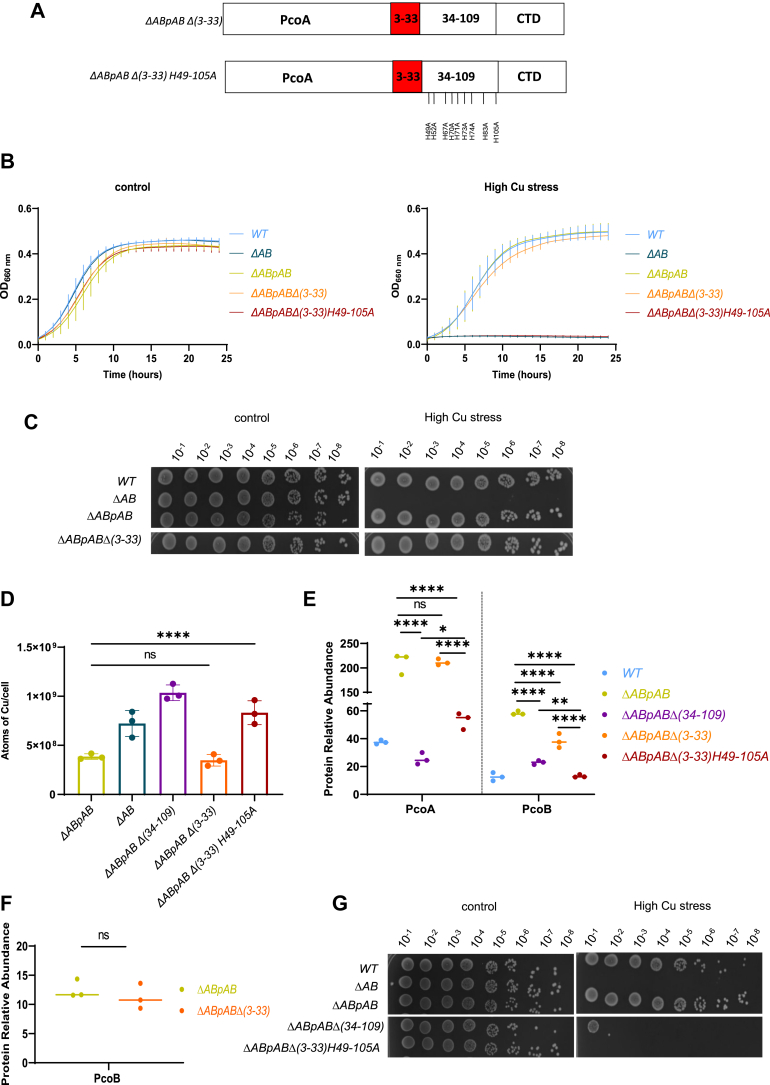
**PcoB harbors an unconventional signal peptide.***A,* schematic representation of the *ΔABpABΔ(3–33)* and *ΔABpABΔ(3–33)H49–105A* mutants. *B,* growth profiles at an absorbance of 660 nm of WT, *ΔAB*, *ΔABpAB*, *ΔABpABΔ(3–33)*, and *ΔABpABΔ(3–33)H49–105A* strains grown in PYE medium (*left*) and in PYE medium supplemented with CuSO_4_ (*right*). *C,* viability assay on PYE plates of WT, *ΔAB*, *ΔABpAB*, and *ΔABpABΔ(3–33)* strains, grown in control and high CuSO_4_ stress conditions. The WT*, ΔAB,* and *ΔABpAB* have been reused from Figure 1*D* because all the strains from Figure 6*C* originate from the same plate as the strains from Figure 1*D*. *D,* number of Cu atoms per cell in the *ΔABpAB, ΔAB, ΔABpABΔ(34–109), ΔABpABΔ(3–33)*, and *ΔABpABΔ(3–33)H49–105A* strains exposed to 175 μM CuSO_4_ for 5 min. Individual values, mean ± SD, and at least three biological replicates are represented. Values for the *ΔABpAB, ΔAB*, and *ΔABpABΔ(34–109)* are the same as in Fig. 1E as it is a unique dataset. *E,* normalized spectrum counts of PcoA and PcoB peptides in the WT*, ΔABpAB*, *ΔABpABΔ(34–109)*, *ΔABpABΔ(3–33),* and *ΔABpABΔ(3–33)H49–105A* strains grown in PYE medium, measured by LC–MS. Individual values and means represented. *p* Values were calculated using ANOVA combined with Dunnett's multiple comparison test (∗*p* < 0.05, ∗∗*p* < 0.01, ∗∗∗*p* < 0.001, and ∗∗∗∗*p* < 0.0001) (Table S1). *F,* PcoB abundance measured by LC–MS in OM extracts of the *ΔABpAB* and *ΔABpAB Δ(3–33)* strains grown in PYE medium. *p* Values were calculated using an unpaired *t* test; ∗*p* < 0.05 (Table S1). *G,* viability assay on PYE plates of *WT*, *ΔAB*, *ΔABpAB*, *ΔABpABΔ(34–109)*, and *ΔABpABΔ(3–33)H49–105A* strains, grown in control and high CuSO_4_ stress conditions. OM, outer membrane; PYE, peptone yeast extract.

The presence of eight His residues within the predicted SP is puzzling and not observed in the other PcoB orthologs (Fig. 3*A*). To address this peculiarity, we constructed the *ΔABpABΔ(3–33)* mutant, lacking the putative SP sequence (Fig. 6*A*). This *ΔABpABΔ(3–33)* mutant displays a *ΔABpAB*-like Cu resistance in both liquid medium (Fig. 6*B*) and on plate (Fig. 6*C*). Furthermore, this mutation does not impact Cu content (Fig. 6*D*) and PcoA level (Fig. 6*E*) but reduces PcoB level in the periplasmic/OM fraction, even if it remains at a much higher level than in the WT strain (Fig. 6*E*). PcoB levels measured in purified OM fraction from the *ΔABpABΔ(3–33)* mutant and the control *ΔABpAB* strains indicate that the PcoB NTD_3–33_ is not required to address PcoB at the OM (Fig. 6*F*) and prompted us to question its role as a canonical SP. To test this hypothesis, we repeated the LC–MS analysis on a periplasmic/OM fraction, combining trypsin and GluC peptidase, the latter cleaving after glutamate I and aspartate (D) residues, potentially generating peptides within the PcoB (3, 4, 5, 6, 7, 8, 9, 10, 11, 12, 13, 14, 15, 16, 17, 18, 19, 20, 21, 22, 23, 24, 25, 26, 27, 28, 29, 30, 31, 32, 33) region. Indeed, the trypsin peptidase (used to digest the proteins before the LC–MS) cleaves after arginine (R) and lysine (K), and the first cleavage site within PcoB aligns with the end of the putative SP (R_33_), which is not optimal for generating peptides within the SP sequence for detection and sequencing. This adapted digestion revealed the presence of the 9 to 33 peptide in the mature form of PcoB (Fig. S5), reinforcing the idea that PcoB does not harbor a conventional SP in *C. vibrioides*, which contrasts with most of the PcoB homologs analyzed in this study, but *Hyphomonas* sp., predicted to harbor a conventional SP by SignalP.

To address the role of the eight His residues located within the PcoB NTD_3–33_, we generated the *ΔABpABΔ(3–33)H49–105A* mutant, which combines the nine His to Ala point mutations of the PcoB NTD_34–109_ to the loss of the eight His located in the PcoB NTD_3–33_ (Fig. 6*A*). This mutant phenocopies the *ΔAB* mutant in terms of Cu sensitivity in liquid medium and on plate (Fig. 6, *B* and *G*) and Cu content (Fig. 6*D*). The *ΔABpABΔ(3–33)H49–105A* mutant also displayed a significant decrease of PcoB level compared with the *ΔABpABΔ(34–109)* and the *ΔABpAB Δ(3–33)* (Fig. 6*E*). However, the PcoA level is significantly different between the *ΔABpABΔ(34–109)* mutant and the *ΔABpABΔ(3–33)* mutant, suggesting that PcoA stabilization by PcoB does not entirely rely on the His residues present in PcoB NTD.

Altogether, our data highlight the role of the PcoB IDR in the stability and function of PcoA and PcoB and in turn in Cu resistance. They also reveal that PcoB IDR is quite tolerant to sequence and size change, if a minimal set of His residues is present.

## Discussion

Cu efflux is a central strategy used by organisms to resist Cu toxicity. In the Gram-negative aquatic alphaproteobacterium *C. vibrioides*, a chromosomally encoded operon supports Cu homeostasis *via* PcoA and PcoB, responsible for Cu oxidation and efflux, respectively (18). While the role of PcoA is established, the mechanism of PcoB-mediated Cu efflux remains poorly understood despite its key role in Cu resistance in several bacterial species, such as *E. coli* and *A. baumannii* (16, 17).

The predicted 3D structure of PcoB reveals a 12-stranded antiparallel β-barrel preceded by a highly flexible, intrinsically disordered NTD comprising 109 residues (19). IDRs are widespread across all domains of life and are crucial for various cellular functions, including signaling, protein interactions, and transcriptional regulation (24).

### Functional role of the PcoB NTD

Our findings demonstrate that a *ΔABpABΔ(34–109)* mutant, lacking most of PcoB NTD, displays an increased Cu sensitivity and intracellular Cu accumulation, highlighting the NTD’s critical role in Cu efflux.

The His- and Met-rich motifs 29HAHH32, 49HAGH52, and 67HAGHHMHH74—within residues 34 to 109—resemble the amino-terminal Cu(II)- and nickel (Ni) (II)-binding (ATCUN) motif NH_2_–X–X–H, typically found in proteins that bind Cu and Ni (25). Accordingly, site-directed mutagenesis of the nine His or of the four Met in this region disrupts Cu efflux. Furthermore, swapping or cropping the 34 to 109 region with the corresponding region from *E. coli* PcoB—containing a minimal set of His and Met residues—retains functionality, suggesting these residues are essential.

A comparable system is seen in the human Cu(I) ion transporter hCtr1, where His- and Met-rich motifs in the disordered NTD aid in Cu acquisition through an energy-independent diffusion process (26).

In Gram-negative bacteria, metal efflux typically relies on tripartite HME–RND complexes energized by the PMF. However, as an OM protein lacking known energy sources such as ATP, GTP, or ion gradients, the energy-independent function of PcoB raises questions. One plausible mechanism is a diffusion-based, entropy-driven process similar to the secretion of the curli-forming CsgA by the CsgG/E/F system in *E. coli* (27), wherein the dynamic PcoB NTD would form a transient pocket for Cu trapping and release *via* Brownian motion.

IDPs often undergo disorder-to-order transitions upon interaction with binding partners (24). In hCtr1, Cu(I) binding induces a transition from disorder to a β-conformation, whereas interaction with lipids promotes α-helical structure formation (28, 29). This suggests that PcoB’s disordered NTD maintains its flexibility through its His residues and adopts a more structured, but transient, state upon Cu binding, facilitating transport.

### NTD contributions to PcoB and PcoA stability

PcoB protein levels were significantly reduced in the *ΔABpABΔ(34–109)* mutant, and even more so in the *ΔABpABΔ(3–33)H49–105A* variant lacking all His in the NTD, suggesting that a minimal number of His is critical for PcoB stability. This effect is not because of altered *pcoB* transcription or PcoB half-life.

The four-base overlap between *pcoA* and *pcoB* ORF supports a model of translational coupling. The present study shows that PcoA stability is also compromised in the absence of PcoB NTD, indicating a functional interplay between PcoA and PcoB. This may involve direct interaction, with the disordered NTD serving as a docking site for PcoA, potentially facilitating Cu(II) transfer from PcoA to PcoB, while stabilizing PcoA.

IDPs often form transient yet specific interactions with globular proteins, involving disorder-to-order transitions within 10 to 50 amino acid segments (30, 31, 32). This mechanism may underline the interaction between PcoB and PcoA.

### Unconventional SP in *Caulobacter* PcoB

As an OM protein, PcoB would be expected to possess a cleavable SP for translocation *via* the Sec pathway (33). However, our computational and experimental analyses indicate that *C. vibrioides* PcoB lacks a classical SP.

This aligns with the phenomen on of “nonclassical secretion,” first described in eukaryotes for proteins such as interleukin-1β and thioredoxin (34, 35), and later observed in bacteria, including secretion of GlnA and SodA without Sec or Tat SPs (36, 37).

In *E. coli*, the HybC subunit is cotransported with its Tat-SP-carrying partner HybO *via* “a piggybg” mechanism (38). Given the potential interaction between PcoA and PcoB, one could hypothesize a similar mechanism where PcoB might undergo translocation through a hitchhiking system, potentially coupled to PcoA through the Tat system. However, the presence of PcoB in the periplasmic/OM fraction of the *ΔABpB* mutant lacking PcoA suggests that PcoB translocation occurs independently.

In conclusion, our study reveals an unprecedented role of an IDR in Cu efflux and protein stabilization, while also challenging the traditional paradigm of SP-dependent OM translocation in Gram-negative bacteria.

## Experimental procedures

### Strains and plasmids

The *C. vibrioides NA1000* (WT) strain was grown at 30 °C, under moderate shaking, in PYE (Poindexter; 1981) medium, supplemented with 5 μg/ml kanamycin, 15 μg/ml nalidixic acid, 100 μg/ml chloramphenicol, and/or CuSO_4_.5H_2_O when required. Exponentially growth cultures were used for all experiments. Plasmids were mobilized from a *DH10B E coli* strain into *C. vibrioides* by triparental mating. Strains and plasmids are listed in Table S2.

#### Construction of the chromosomal mutant

To perform the NTD deletion on the chromosome, the upstream and downstream regions of the *pcoB* gene were amplified separately by Q5 PCR in the WT strain. The full-length PCR fragment covering the upstream and downstream regions was amplified by overlap-extension PCR method described (39) and has then been inserted into an EcoRV linearized suicide vector pNPTS138. The ligation product was then transformed into a DH10B *E coli* strain. A triparental mating was performed between the *E. coli* strain containing the appropriate pNPTS138, the S17-1 helper strain of *E. coli*, and the appropriate strain of *C. vibrioides*. A first selection for kanamycin-resistant clones was conducted to select bacteria that integrated the pNPTS138 by homologous recombination. Selection for a second recombination event was then performed by cultivating the clones in PYE medium before plating them on PYE + 3% sucrose plates. Strains that lost the plasmid could either be a knockout strain for the gene of interest or a WT strain. The genotype of the clones was determined by PCR.

#### Construction of the plasmidic mutants

The sequence of the *pcoAB* operon was amplified by Q5 PCR in the WT strain, and the PCR product was inserted into an EcoRV-linearized pSK plasmid. This construction served as a backbone for the different constructions. The NTD of *E. coli* was amplified using a Gbloc with the N_E.c_ sequence (270 bp). The different mutations (domain and subdomain deletions, domain swapping, and directed point mutations) were performed using the overlap-extension PCR as described (39, 40). The newly amplified insert with the mutations was inserted into an EcoRV-linearized pSK plasmid and then excised with appropriate restriction enzymes and inserted into a pMR10 plasmid restricted with the same restriction enzymes. This pMR10 plasmid was transformed into a DH10B *E coli* strain. A triparental mating was finally performed between the *E. coli* strain containing the appropriate pMR10, the S17-1 helper strain of *E. coli*, and the appropriate strain of *C. vibrioides*. The clones growing on PYE + kanamycin plates were tested by PCR to confirm the presence of the gene of interest in the pMR10.

The schemes for the constructions and the sequences of the primers are provided in Fig. S6 and Table S3, respectively.

### Growth curves

Bacterial cultures in the exponential growth phase (absorbance of 0.4–0.6 at 660 nm) were diluted in PYE medium to a final absorbance of 0.05 at 660 nm and inoculated in 96-well plates with appropriate CuSO_4_ concentrations when required. Bacteria were then grown for 24 h at 30 °C under continuous shaking in an Epoch 2 Microplate Spectrophotometer from BioTek, and absorbance at 660 nm was measured every 10 min.

### Viability assay

Bacterial cultures in the exponential growth phase (absorbance of 0.4–0.6 at 60 nm) were diluted in PYE medium to a final absorbance of 0.1 at 660 nm. Ten-fold serial dilutions up to 10^-8^ (in PYE) were prepared in 96-well plates, and drops of 5 μl of each dilution were spotted on PYE and PYE Cu plates using an automatic multichannel. Plates were incubated for 48 h at 30 °C, and pictures were taken with the Amersham Imager 600 (GE Healthcare LifeSciences).

### Inductively coupled plasma optical emission spectroscopy

Cultures of *C. vibrioides* cells (15 ml) were grown up to exponential phase (absorbance of 0.5 at 660 nm). A 5-min treatment with 175 μM final CuSO_4_ concentration was applied, and the cultures were then centrifuged at 8500 rpm for 10 min at 4 °C. The cells were then fixed for 20 min on ice in 2% paraformaldehyde and then washed three times with an ice-cold wash buffer (10 mM Tris–HCl [pH 6.8]and 100 μM EDTA). For a total fraction, where the cytoplasm and periplasm are not separated, the pellet is resuspended in 2 ml of milliQ H_2_O and then lysed with the cell disrupter (Cell Disruption System; One-Shot Model, Constant) at 2.48 kPa. A final centrifugation at 10,000 rpm is performed for 10 min, and 1.6 ml of the supernatant is mixed with 1 ml HNO_3 (_5%) and 2.4 ml milliQ H_2_O for a final volume of 5 ml and a final concentration of 1 M HNO_3_. Samples were finally analyzed by ICP-OES with Optima 8000 ICP-OES (PerkinElmer), and cellular metal concentrations were calculated using the following formula as in (18):

$$\text{Atoms}\;\text{of}\;\text{Cu}/\text{cell}=\frac{\frac{\text{Amount}\;\text{of}\;\text{metal}\;\left(\text{mg}\right)}{\text{Molecular}\;\text{weight}\;\text{of}\;\text{matal}\;\left(\text{mg}/l\right)}\times \text{Avogadro}\;\text{constant}\;\left(6.022*10^{23}\right)}{\text{Number}\;\text{of}\;\text{bacteria}}$$

### Mass spectrometry

#### Periplasmic/OM sample preparation

Cultures of *C. vibrioides* cells (15 ml) were grown up to exponential phase (absorbance at 660 nm = 0.4–0.6). The cultures were then centrifuged at 8000 rpm for 10 min at 4 °C (trash supernatant) and washed three times with an ice-cold wash buffer (10 mM Tris–HCl [pH 6.8], 100 μM EDTA). The pellets were then incubated in 2 ml, V/v of Zwittergent 0.25%, and Zwittergent buffer (0.2 M Tris–HCl [pH 7.6]) for 10 min at room temperature and centrifuged for 15 min at 19,000*g*. The supernatant, containing the periplasmic/OM components, was separated from the cytoplasmic pellet and collected as samples for LC–MS analysis.

#### OM sample preparation

The OM fraction of *C. vibrioides* was harvested by ultracentrifugation after sodium lauryl sarconisate and sodium carbonate treatment as described (41). The cell culture (400 ml) was grown in PYE at 30°C to an absorbance of 0.4 at 660 nm. Bacteria were centrifuged for 10 min at 4000*g* at 4 °C and washed three times in 50 ml of 50 mM (pH 8) ammonium bicarbonate. Cells were finally resuspended in 5 ml ammonium bicarbonate and sonicated on ice (20 rounds of 5 s sonication at maximum intensity). The lysate was centrifuged for 20 min at 12,000*g* at 4 °C, and the pellet containing the unbroken cells and debris was discarded. The supernatant was then ultracentrifuged for 40 min at 100,000*g* to dissociate the cytoplasmic fraction (supernatant) from the total membrane fraction (pellet). The pellet was then resuspended in 1 ml of 1% sodium lauryl sarconisate incubated 30 min at room temperature and centrifuged for another 40 min at 100,000*g* and 4 °C. The subsequent supernatant contains the solubilized inner membrane and was discarded. The pellet containing the OM fraction was washed in 1 ml of 2.5 M NaBr and incubated for 30 min on ice. It was then ultracentrifuged for 40 min at 100,000*g* at 4 °C. The supernatant was discarded, and the pellet was incubated for 1 h in 1 ml of 100 mM Na_2_CO_3_ for further enrichment of the OM fraction, before being spun again for 40 min at 100,000*g* at 4 °C. The OM fractions were collected as samples for LC–MS analysis.

#### Liquid chromatography–mass spectrometry

The samples were treated using the optimized filter-aided sample preparation protocol (42). Briefly, the samples were loaded onto Millipore Microcon 30 MRCFOR030 Ultracel PL-30 filters that have been rinsed and washed beforehand with 1% formic acid (FA) and 8 M urea buffer (8 M urea in 0.1 M Tris buffer at pH 8.5), respectively. The proteins on the filter were then exposed to a reducing agent (dithiothreitol) and then alkylated with iodoacetamide. The proteins were then finally digested overnight at 25 °C with trypsin or trypsin/Glu-C (trypsin in 1/50 in ABC buffer; Glu-C in phosphate buffer 50 mM [pH 7.4]). The final step of digestion is to transfer proteins in 20 μl of 2% acetonitrile and 0.1% FA in an injection vial for reverse-phase chromatography. The digest was analyzed using nano-LC–ESI–MS/MS timsTOF Pro (Bruker) coupled with an UHPLC nanoElute (Bruker). Peptides were separated on a 75 μm ID, 25 cm C18 column with integrated Captive Spray insert (Aurora, Ion Opticks) at a flow rate of 200 nl/min at 50 °C. LC mobile phase A was water with 0.1% FA (v/v), and mobile phase B was acetonitrile with FA 0.1% (v/v). Samples were loaded directly on the analytical column at a constant pressure of 800 bars. The digest (1 μl) was injected, and the organic content of the mobile phase was increased linearly from 2% B to 15% in 22 min, 15% B to 35% in 38 min, and 35% B to 85% in 3 min. Data acquisition on the tims TOF Pro was performed using Hystar 5.1 and tims Control 2.0. tims TOF Pro data were acquired using 100 ms TIMS accumulation time and mobility (1/K0) range from 0.6 to 1.6 Vs/cm^2^. MS1 mass range was 100 to 1700 *m/z*. Mass-spectrometric analysis was carried out using the parallel accumulation serial fragmentation acquisition method (43). One MS spectrum was followed by 10 parallel accumulation serial fragmentation MSMS spectra per total cycle of 1.1 s. Maximum charge was set to 5. Precursor repetitions were set to 20,000 for target intensity and 2500 for the threshold. Exclusion was set to active with a release after 0.4 min. Collision energy was 20 eV for ion mobility of 0.6 V s/cm^2^ and 59 for ion mobility of 1.6 V s/cm^2^. All MS/MS samples were analyzed using Mascot (Matrix Science; version 2.8.1). Mascot was set up to search the *C. vibrioides* NA1000_190306 database from UniRef 100 and Contaminants_20190304 database assuming the digestion enzyme trypsin/GluC. Mascot was searched with a fragment ion mass tolerance of 0.050 Da and apparent ion tolerance of 15 PPM. Carbamidomethyl of cysteine was specified in Mascot as a fixed modification. Oxidation of Met and acetyl of the N terminus was specified in Mascot as variable modifications. Scaffold (version Scaffold_5.1.1; Proteome Software, Inc) was used to validate MS/MS-based peptide and protein identifications. Spectral counting was used for relative quantification. Peptide identifications were accepted if they could be established at greater than 97.0% probability to achieve a false discovery rate less than 1.0% by the Percolator posterior error probability calculation (44). Protein identifications were accepted if they could be established at greater than 50.0% probability to achieve a false discovery rate less than 1.0% and if they contain at least one identified peptide. Protein probabilities were assigned by the Protein Prophet algorithm (45). Proteins that contained similar peptides and could not be differentiated based on MS/MS analysis alone were grouped to satisfy the principles of parsimony. Proteins sharing significant peptide evidence were grouped into clusters.

Given that we are working with different lengths of the PcoB protein, only peptides corresponding to the CTD (residues 111–302) were included in the quantification analysis. Peptides from the NTD were excluded from the Scaffold file to ensure consistent comparison of peptide counts.

#### Western blotting

Exponentially growing cells were collected and resuspended in an SDS-PAGE loading buffer, with the pellet volumes adjusted to achieve an absorbance of 1 at 660 nm, ensuring protein content normalization. The protein samples were then boiled and separated on 12% sodium dodecyl sulfate-polyacrylamide gels before being electrotransferred to a nitrocellulose membrane. Subsequently, the nitrocellulose membranes were probed with polyclonal rabbit anti-PcoA (1:5000 dilution) and anti-PcoB (1:1000 dilution). A polyclonal goat anti-rabbit immunoglobulins/horseradish peroxidase secondary antibody at a dilution of 1:10,000 (DAKO) was employed.

Cy5 fluorescence was used as a loading control by adding 1 μl of Amersham Cy5 dye to the protein samples, followed by incubation for 30 min at room temperature and heating for 5 min at 95 °C. The Cy5 fluorescence was detected using the Amersham Imager 600.

#### RT–qPCR

Bacteria were grown in PYE up to an absorbance of 0.4 at 660 nm before incubation at 30 °C under agitation. Bacteria were recovered by centrifugation, and pellets were flash-frozen until resuspension in 40 μl of a 20 mg/ml proteinase K solution (Avantor) with 1 μl of undiluted Ready-Lyse Lysozyme Solution (Lucige), and lysis was allowed to proceed for 10 min in a shaking incubator at 37 °C and 600 rpm. Total RNA was retrieved from the cell suspensions using TriPure isolation reagent and procedure as described by the manufacturer (Roche). RNA (2 μg) isolated from *C. vibrioides* was incubated with DNase I (Thermo Scientific) for 30 min at 37 °C. DNase I was then inactivated with 50 mM EDTA for 10 min at 65 °C. Subsequently, RNA was subjected to reverse transcription using MultiScribe Reverse Transcriptase (Applied Biosystems) with random primers (as described by the manufacturer). A total of 300 ng of complementary DNA was mixed with Takyon No Rox SYBR MasterMix dTTP Blue (Eurogentec), and the appropriate primer sets were used for quantitative PCR in LightCycler96 (Roche). Forty-five PCR cycles were performed (95 °C for 10 s, 60 °C for 10 s, and 72 °C for 10 s). Primer specificity was checked by melting curve analysis. Relative gene expression levels between different samples were calculated with the 2−ΔΔCt method using the rpoD gene as a reference. Four biological replicates were analyzed for each sample.

The sequences of the primers are provided in Table S4.

#### Protein half-life

Exponentially growing cells (absorbance = 0.4–0.6 at 660 nm) were treated with 100 μg/ml chloramphenicol to arrest protein synthesis. Samples were collected at 1 h, 2 h, and 4 h after the antibiotic treatment. Pellet volumes were adjusted to achieve an absorbance of 1 at 660 nm in 2 ml of milliQ H_2_O, ensuring protein content normalization at each time point. Total fraction of each time point was generated using the cell disrupter (Cell Disruption System; One-Shot Model, Constant) at 2.48 kPa. A final centrifugation at 10,000 rpm is performed for 10 min, and 1.5 ml of the supernatant was collected. Samples were analyzed with LC–MS.

#### Statistical analysis

Statistical analyses were performed when required. The data were analyzed with one-way ANOVA combined with Dunnett's multiple comparisons test. *p* Values below 0.05, 0.01, 0.001, and 0.0001 are represented by ∗, ∗∗, ∗∗∗, and ∗∗∗∗, respectively. A *t* test was performed when needed.

A linear regression statistical test was performed for the protein half-life experiment.

#### Sequence-based bioinformatic predictions

A multiple sequence alignment of PcoB protein from *C. vibrioides* (Uniprot ID: A0A0H3C699) and 13 homologs from other bacterial species—*Hyphomonas* sp. (UniProt ID: A0A922YF47), *Sphingobium cupriresistens* (UniProt ID: A0A0J7XK94), *Rhizobium* sp. (UniParcID: UPI0022C4F4F1), *Methylobacterium aquaticum* (UniProt ID: A0A0J6SI10), *Nitrobacter* sp. (National Center for Biotechnology information [NCBI] ID: WP_319798384), *Bordetella bronchiseptica* (UniParcID: UPI00045A6DA4), *Pseudomonas aeruginosa* (UniParcID: UPI00377290C9), *Enterobacter cloacae* (NCBI ID: WP_185812793), *Salmonella enterica* (UniParcID: UPI001DC5DD2D), *Klebsiella pneumoniae* (NCBI ID: WP_183406058), *Klebsiella oxytoca* (UniProt ID: A0A6C0L247), *A. baumannii* (UniProt ID: A0A142G3V9), and *E. coli* (UniProt ID: Q47453)—was first performed using the online MAFFT tool (https://www.ebi.ac.uk/jdispatcher/msa). The results were visualized using Jalview.

Intrinsic disorder predictions were carried out using the Rapid Intrinsic Disorder Analysis Online (RIDAO) platform (46), which integrates seven well-established per-residue disorder predictors: PONDR VLXT, VL3, VSL2B, PONDR FIT, IUPred-Short, IUPred-Long, and ANCHOR2. The mean disorder profile (σ(MDP)), calculated as the average of the scores from the seven core algorithms mentioned previously, was used to generate the disorder plots. Residues with a mean score above 0.5 were considered disordered, whereas those with a score below that threshold were classified as structured.

## Data availability

All data are contained within the article and the Supporting Information section. The raw data can be shared upon request to jean-yves.matroule@unamur.be.

## Supporting information

This article contains supporting information.

## Conflict of interest

The authors declare that they have no conflicts of interest with the content of this article.
