# Supplementary material for: Key roles in copper efflux and protein homeostasis of the intrinsically disordered region of a bacterial outer membrane channel
Source: J Biol Chem. 2025 Sep 1;301(10):110670. doi: 10.1016/j.jbc.2025.110670 (PMC12509749; doi:10.1016/j.jbc.2025.110670)
Supplement: Supplementary figures [file mmc1.pdf]

Figure S1

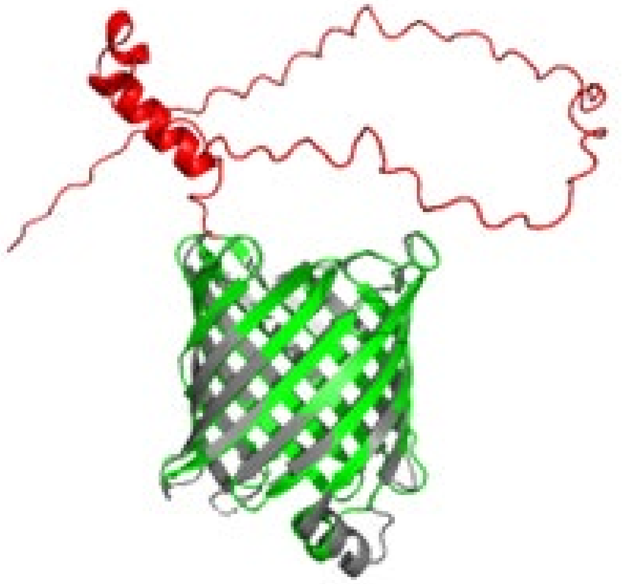

Fig S1 – Structural alignment of PcoB with the crystal structure 7PGE. The crystal structure 7PGE is shown in gray, the β-barrel region of PcoB (residues 110–302) is colored in green, and the N-terminal disordered region of PcoB is highlighted in red. The alignment yielded an RMSD of 0.632 Å over 131 Cα atoms, indicating high structural similarity between the β-barrel domains.

Figure S2

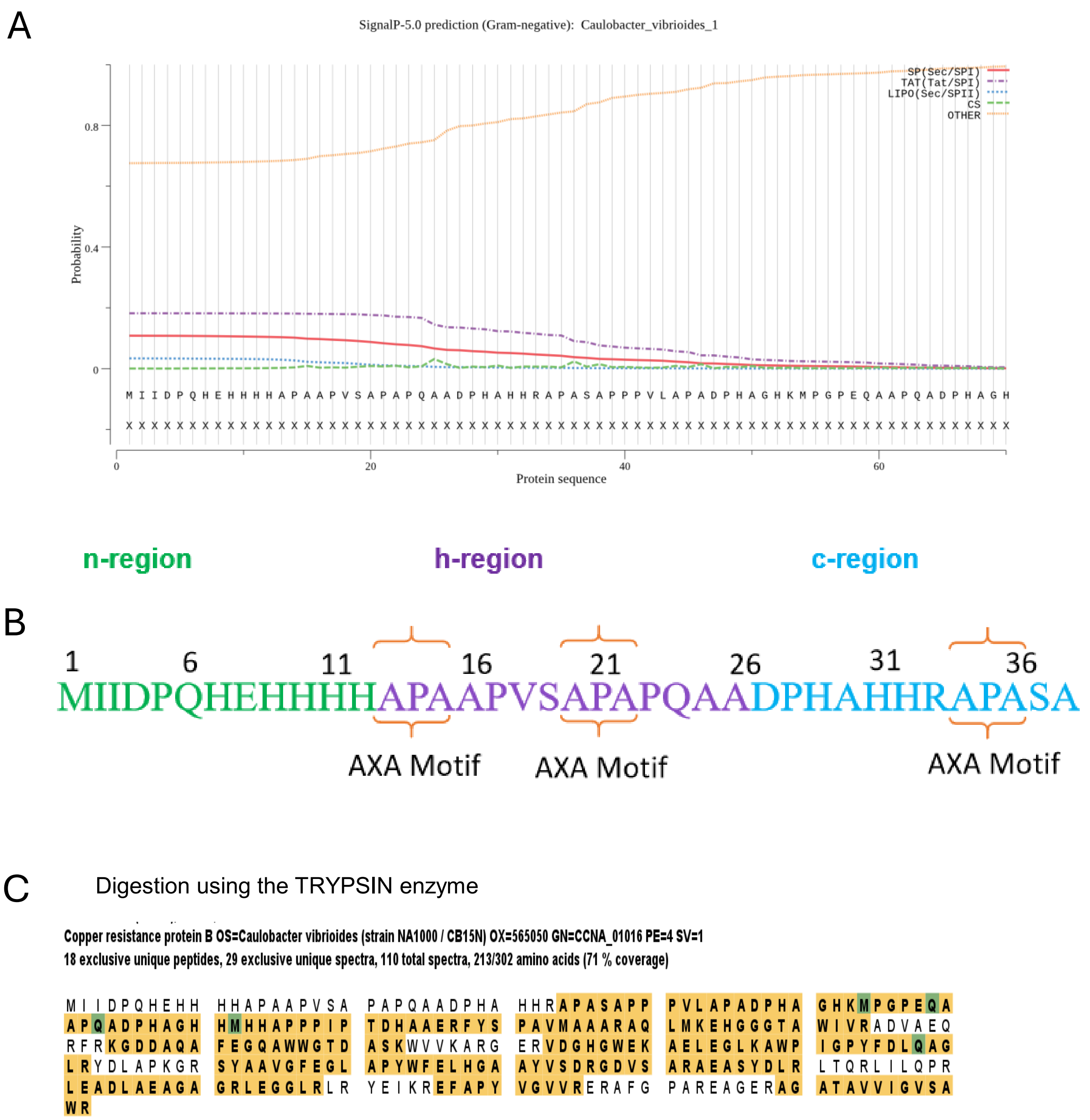

Figure S2. Prediction of a signal peptide in PcoB. A. SignalP5 analysis of PcoB protein sequence. **B.** Manual analysis of PcoB protein sequence. **C.** Sequenced peptide of the Trypsin-digested PcoB from a periplasm/OM fractionate.

Figure S3

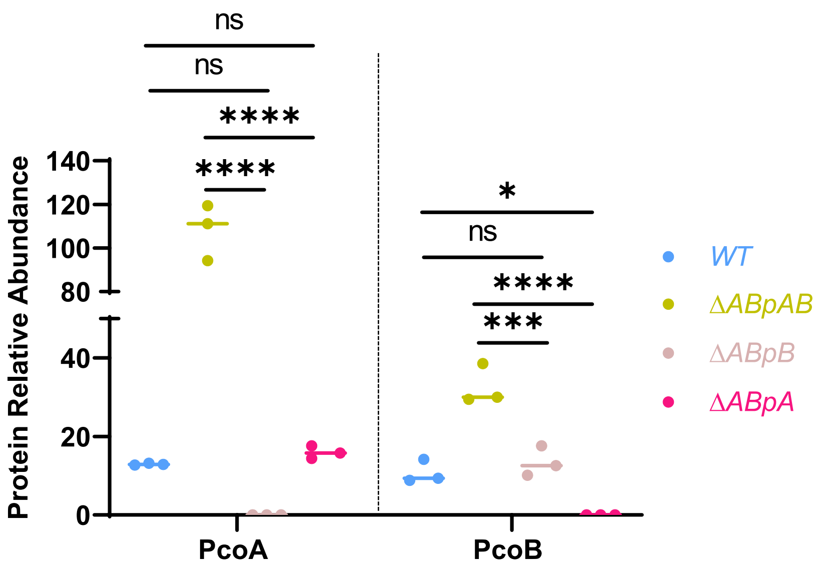

Figure S3. PcoA and PcoB interdependence. Normalized spectrum counts of PcoA and PcoB peptides in the WT, *ΔABpAB, ΔABpA and ΔABpB* strains grown in PYE medium, measured by LC-MS. Individual values and means represented. Values for the WT, ΔABpAB are the same as in Fig.4E as it is a unique data set. p values were calculated using ANOVA combined with Dunnett's multiple comparison test (*$p < 0.05$, ** $p < 0.01$, *** $p < 0.001$ and ****$p < 0.0001$) **(Table S1).**

Figure S4

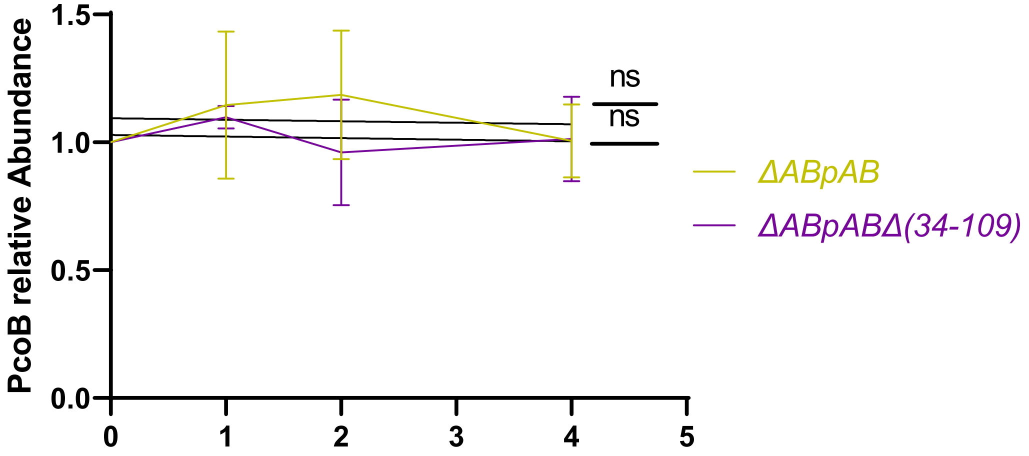

Figure S4. PcoB half-life. PcoB abundance measured by LC/MS in total cell extracts obtained 0 h, 1 h, 2 h and 4 h after chloramphenicol treatment of the ΔABpAB and ΔABpABΔ(34-109) strains grown in PYE medium. *p* values were calculated using a linear regression statistical test; $*p < 0.05$ **(Table S1)**.

Figure S5

Digestion using trypsine+ GluC enzymes

Copper resistance protein B OS=Caulobacter vibrioides (strain NA1000 / CB15N) OX=565050 GN=CCNA_01016 PE=4 SV=1
16 exclusive unique peptides, 24 exclusive unique spectra, 65 total spectra, 180/302 amino acids (60 % coverage)

```
MIIDPQHEHH HHAPAAPVSA PAPQAADPHA HHRAPASAPP PVLAPADPHA GHKMPGPEQA
APQADPHAGH HMHHAPPPIP TDHAAERFYS PAVMAAARAQ LMKEHGGGTA WIVRADVAEQ
RFRKGDDAQA FEGQAWWGTD ASKWVVKARG ERVDGHGWEK AELEGLKAWP IGPYFDLQAG
LRYDLAPKGR SYAAVGFEGL APYWFELHGA AYVSDRGDVS ARAEASYDLR LTQRLILQPR
LEADLAEAGA GRLEGGLRLR YEIKREFAPY VGVVRERAFG PAREAGERAG ATAVVIGVSA
WR
```

Figure S5. The predicted signal peptide is not cleaved. Sequenced peptide of the trypsin/GluC-digested PcoB from a periplasm/OM fractionate

Figure S6 (1/2)

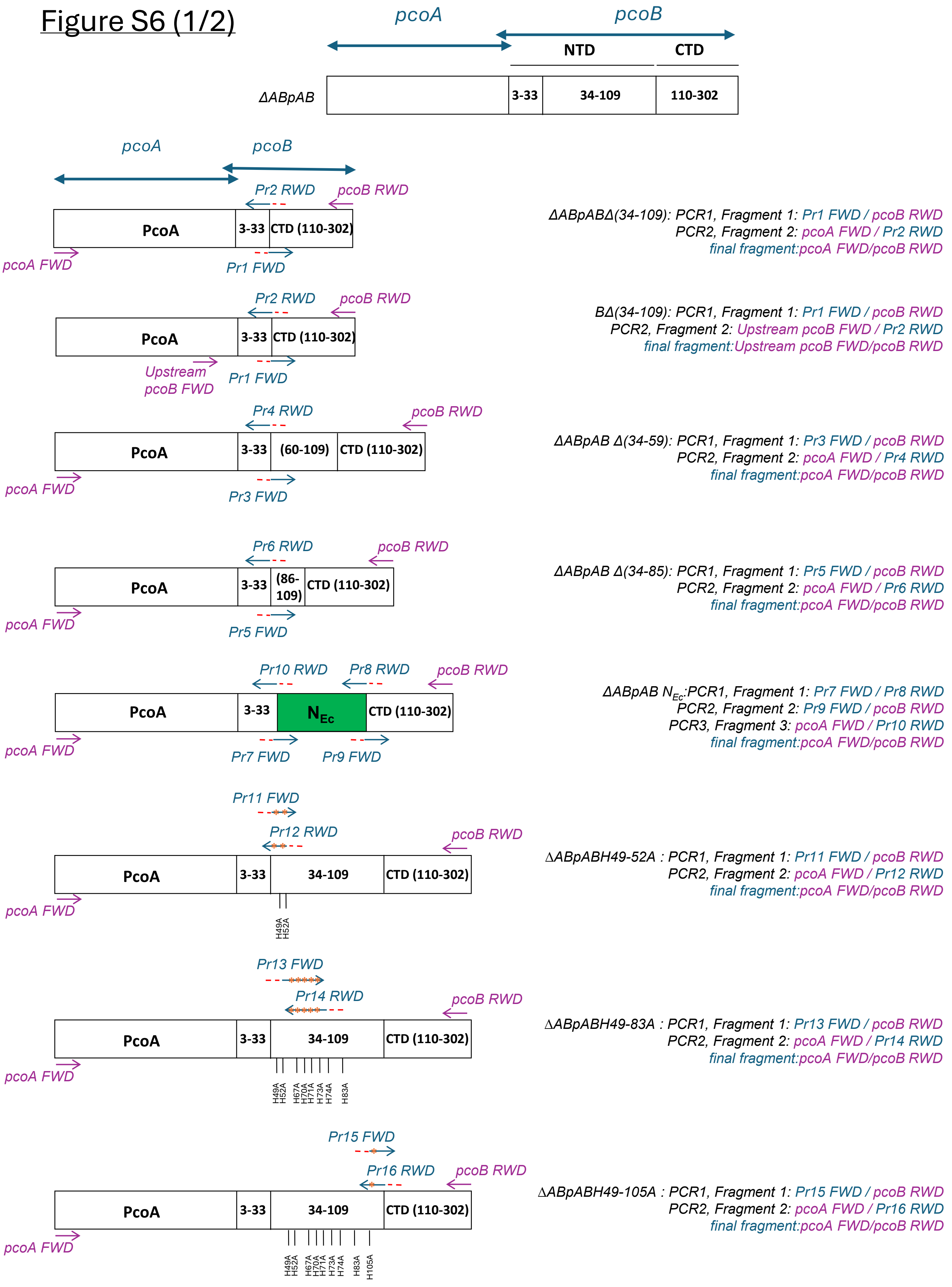

pcoA
pcoB
NTD
CTD
ΔABpAB
3-33
34-109
110-302
pcoA
pcoB
Pr2 RWD
pcoB RWD
PcoA
3-33
CTD (110-302)
pcoA FWD
Pr1 FWD
ΔABpABΔ(34-109): PCR1, Fragment 1: Pr1 FWD / pcoB RWD
PCR2, Fragment 2: pcoA FWD / Pr2 RWD
final fragment:pcoA FWD/pcoB RWD
Pr2 RWD
pcoB RWD
PcoA
3-33
CTD (110-302)
Upstream pcoB FWD
Pr1 FWD
BΔ(34-109): PCR1, Fragment 1: Pr1 FWD / pcoB RWD
PCR2, Fragment 2: Upstream pcoB FWD / Pr2 RWD
final fragment:Upstream pcoB FWD/pcoB RWD
Pr4 RWD
pcoB RWD
PcoA
3-33
(60-109)
CTD (110-302)
pcoA FWD
Pr3 FWD
ΔABpAB Δ(34-59): PCR1, Fragment 1: Pr3 FWD / pcoB RWD
PCR2, Fragment 2: pcoA FWD / Pr4 RWD
final fragment:pcoA FWD/pcoB RWD
Pr6 RWD
pcoB RWD
PcoA
3-33
(86-109)
CTD (110-302)
pcoA FWD
Pr5 FWD
ΔABpAB Δ(34-85): PCR1, Fragment 1: Pr5 FWD / pcoB RWD
PCR2, Fragment 2: pcoA FWD / Pr6 RWD
final fragment:pcoA FWD/pcoB RWD
Pr10 RWD
Pr8 RWD
pcoB RWD
PcoA
3-33
N$_{Ec}$
CTD (110-302)
pcoA FWD
Pr7 FWD
Pr9 FWD
ΔABpAB N$_{Ec}$:PCR1, Fragment 1: Pr7 FWD / Pr8 RWD
PCR2, Fragment 2: Pr9 FWD / pcoB RWD
PCR3, Fragment 3: pcoA FWD / Pr10 RWD
final fragment:pcoA FWD/pcoB RWD
Pr11 FWD
Pr12 RWD
pcoB RWD
PcoA
3-33
34-109
CTD (110-302)
pcoA FWD
H49A
H52A
ΔABpABH49-52A : PCR1, Fragment 1: Pr11 FWD / pcoB RWD
PCR2, Fragment 2: pcoA FWD / Pr12 RWD
final fragment:pcoA FWD/pcoB RWD
Pr13 FWD
Pr14 RWD
pcoB RWD
PcoA
3-33
34-109
CTD (110-302)
pcoA FWD
H49A
H52A
H67A
H70A
H71A
H73A
H74A
H83A
ΔABpABH49-83A : PCR1, Fragment 1: Pr13 FWD / pcoB RWD
PCR2, Fragment 2: pcoA FWD / Pr14 RWD
final fragment:pcoA FWD/pcoB RWD
Pr15 FWD
Pr16 RWD
pcoB RWD
PcoA
3-33
34-109
CTD (110-302)
pcoA FWD
H49A
H52A
H67A
H70A
H71A
H73A
H74A
H83A
H105A
ΔABpABH49-105A : PCR1, Fragment 1: Pr15 FWD / pcoB RWD
PCR2, Fragment 2: pcoA FWD / Pr16 RWD
final fragment:pcoA FWD/pcoB RWD

Figure S6 (2/2)

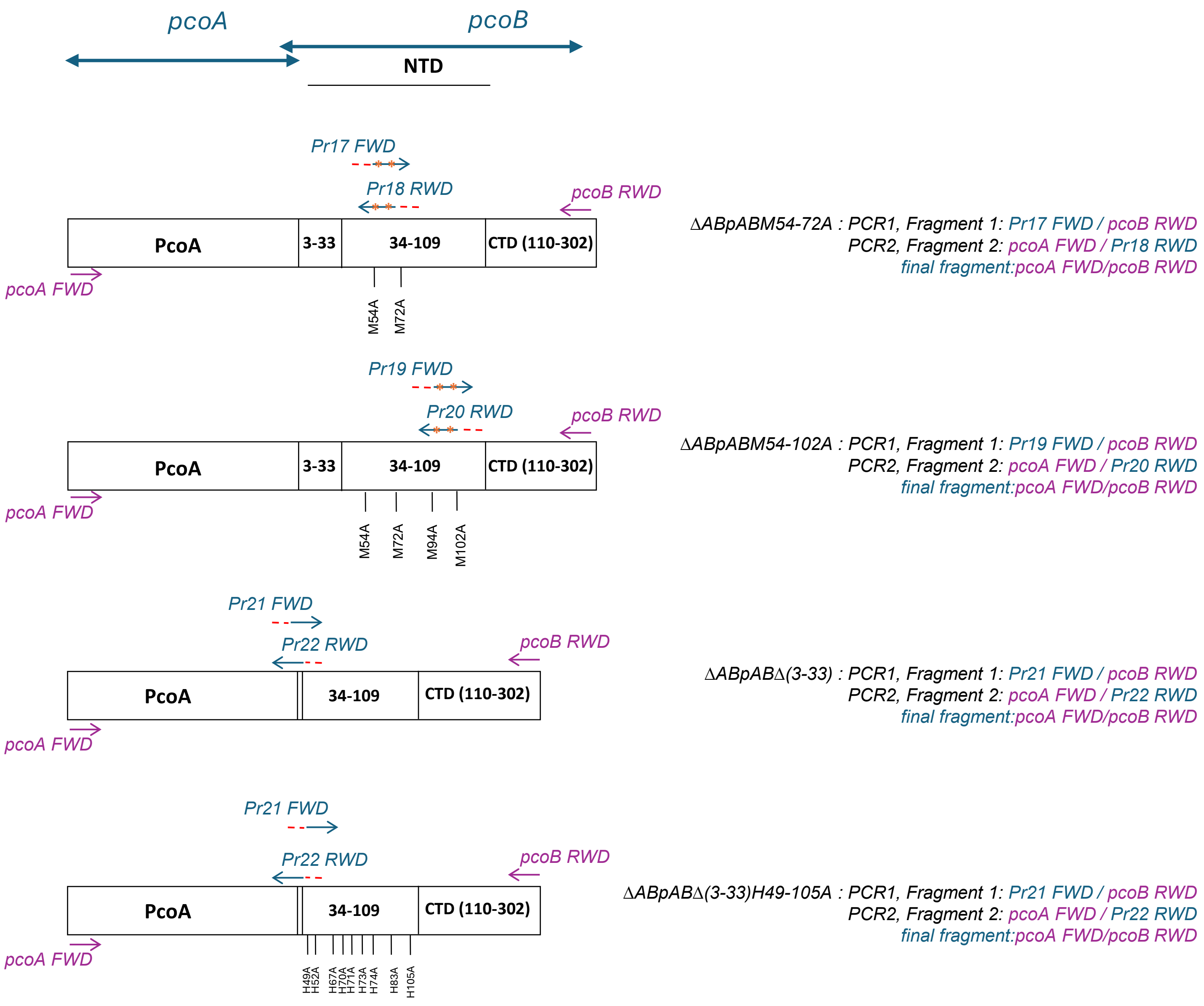

Figure S6. Schematic diagrams of overlap extension PCR's for deletion, insertion and point mutations. Primers for the intermidiate fragments amplification are in blue with red overlapping regions, in purple FWD and RWD primers for the final pcoAB fragment amplification.
