# Supplementary material for: Key roles in copper efflux and protein homeostasis of the intrinsically disordered region of a bacterial outer membrane channel
Source: J Biol Chem. 2025 Sep 1;301(10):110670. doi: 10.1016/j.jbc.2025.110670 (PMC12509749; doi:10.1016/j.jbc.2025.110670)
Supplement: Supplementary tables [file mmc2.pdf]

Table S1: pvalues. One-way ANOVA and t-test were performed using GraphPad Prism.

Fig. 1E ANOVA one way / Dunnett's multiple comparisons test	Summary	Adjusted P Value
ΔAB vs. *WT*	ns	0,2256
ΔAB vs. *ΔABpAB*	***	0,0004
ΔAB vs. *ΔABpAB* Δ(34-109)	**	0,001
ΔAB vs. *B* Δ(34-109)	ns	0,7985

Fig. 2B ANOVA one way / Dunnett's multiple comparisons test	Summary	Adjusted P Value
PcoB		
Δ*ABpAB* vs. WT	****	<0,0001
Δ*ABpAB* vs. Δ*ABpAB*Δ(34-109)	****	<0,0001
Δ*ABpAB* vs. *B*Δ(34-109)	****	<0,0001
WT vs. Δ*ABpAB*Δ(34-109)	ns	0,328
WT vs. *B*Δ(34-109)	ns	0,9665
PcoA		
Δ *ABpAB* vs. WT	****	<0,0001
Δ *ABpAB* vs. Δ *ABpAB* Δ(34-109)	***	0,0001
Δ *ABpAB* vs. BΔ(34-109)	****	<0,0001
WT vs. Δ *ABpAB* Δ(34-109)	ns	0,8871
WT vs. *B*Δ(34-109)	ns	0,8556

Fig. 2C Unpaired t test	Summary	Adjusted P Value
PcoB		
Δ *ABpAB* vs. Δ *ABpAB* Δ(34-109)	ns	0,3264
PcoA		
Δ *ABpAB* vs. Δ *ABpAB* Δ(34-109)	ns	0,0558

Fig. 2D Simple linear regression	Summary	Adjusted P Value
PcoA		
ΔABpAB	ns	0,0582
*ΔABpAB*Δ(34-109)	****	<0,0001

Fig. 4D Unpaired t test	Summary	Adjusted P Value
ΔABpAB vs. ΔABpAB NE.c	ns	0,8192

Fig. 4E ANOVA one way / Dunnett's multiple comparisons test	Summary	Adjusted P Value

PcoB		
Δ *ABpAB* vs. WT	****	<0,0001
Δ *ABpAB* vs. Δ *ABpAB* Δ(34-109)	**	0,0014
Δ *ABpAB* vs. Δ *ABpAB* Δ(34-59)	**	0,0066
Δ *ABpAB* vs. Δ *ABpAB* Δ(34-85)	ns	0,7061
Δ *ABpAB* vs. Δ *ABpAB* NE.c	ns	0,4998
PcoA		
Δ *ABpAB* vs. WT	****	<0,0001
Δ *ABpAB* vs. Δ *ABpAB* Δ(34-109)	****	<0,0001
Δ *ABpAB* vs. Δ *ABpAB* Δ(34-59)	ns	0,9997
Δ *ABpAB* vs. Δ *ABpAB* Δ(34-85)	ns	0,7586
Δ *ABpAB* vs. Δ *ABpAB* NE.c	****	<0,0001

Fig. 4G ANOVA one way / Dunnett's multiple comparisons test	Summary	Adjusted P Value
ΔABpAB vs. *ΔAB*	***	0,0004
ΔABpAB vs. *ΔABpAB* Δ(34-109)	****	<0,0001
ΔABpAB vs. *ΔABpAB* Δ(34-59)	ns	0,3653
ΔABpAB vs. *ΔABpAB* Δ(34-85)	ns	0,6733

Fig. 4E ANOVA one way / Dunnett's multiple comparisons test	Summary	Adjusted P Value
PcoB		
Δ *ABpAB* vs. WT	**	0,0025
Δ *ABpAB* vs. Δ *ABpAB* Δ(34-109)	**	0,0062
Δ *ABpAB* vs. Δ *ABpAB* H49-105A	ns	0,1508
Δ *ABpAB* vs. Δ *ABpAB* M52-102A	*	0,0493
PcoA		
Δ *ABpAB* vs. WT	****	<0,0001
Δ *ABpAB* vs. Δ *ABpAB* Δ(34-109)	***	0,0003
Δ *ABpAB* vs. Δ *ABpAB* H49-105A	ns	0,2768
Δ *ABpAB* vs. Δ *ABpAB* M52-102A	**	0,0039

Fig. 5F ANOVA one way / Dunnett's multiple comparisons test	Summary	Adjusted P Value
ΔABpAB vs. *ΔAB*	***	0,0004
ΔABpAB vs. *ΔABpAB* Δ(34-109)	****	<0,0001
ΔABpAB vs. *ΔABpAB* H49-105A	****	<0,0001
ΔABpAB vs. Δ *ABpAB* M54-102A	****	<0,0001

Fig. 6D ANOVA one way / Dunnett's multiple comparisons test	Summary	Adjusted P Value
ΔABpAB vs. *ΔAB*	***	0,0004
ΔABpAB vs. *ΔABpAB* Δ(34-109)	****	<0,0001
ΔABpAB vs. *ΔABpAB* Δ(3-33)	ns	0,9968
ΔABpAB vs. *ΔABpAB* Δ(3-33) H49-105A	****	<0,0001

Fig 6E ANOVA one way / Dunnett's multiple comparisons test	Summary	Adjusted P Value
PcoB		
Δ *ABpAB* vs. WT	****	<0,0001
Δ *ABpAB* vs. Δ *ABpAB* Δ(34-109)	****	<0,0001
Δ *ABpAB* vs. Δ *ABpAB* Δ(3-33)	****	<0,0001
Δ *ABpAB* vs. Δ *ABpAB* Δ(3-33)H49-105A	****	<0,0001
Δ *ABpAB* Δ(34-109) vs. WT	**	0,004
Δ *ABpAB* Δ(34-109) vs. Δ *ABpAB* Δ(3-33)	***	0,0002
Δ *ABpAB* Δ(34-109) vs. Δ *ABpAB* Δ(3-33)H49-105A	**	0,0056
PcoA		
Δ *ABpAB* vs. WT	****	<0,0001
Δ *ABpAB* vs. Δ *ABpAB* Δ(34-109)	****	<0,0001
Δ *ABpAB* vs. Δ *ABpAB* Δ(3-33)	ns	0,9999
Δ *ABpAB* vs. Δ *ABpAB* Δ(3-33)H49-105A	****	<0,0001
Δ *ABpAB* Δ(34-109) vs. WT	ns	0,4636
Δ *ABpAB* Δ(34-109) vs. Δ *ABpAB* Δ(3-33)	****	<0,0001
Δ *ABpAB* Δ(34-109) vs. Δ *ABpAB* Δ(3-33)H49-105A	*	0,0279

Fig. 6F Unpaired t test	Summary	Adjusted P Value
ΔABpAB vs. Δ*ABpAB*Δ(3-33)	ns	0,4526

Fig S3 ANOVA one way / Dunnett's multiple comparisons test	Summary	Adjusted P Value
PcoB		
Δ *ABpAB* vs. WT	***	0,0002
Δ *ABpAB* vs. Δ*ABpB*	***	0,0004
Δ *ABpAB* vs. Δ*ABpA*	****	<0,0001
WT vs. Δ*ABpB*	ns	0,5724
WT vs. Δ*ABpA*	*	0,0134
PcoA		
Δ *ABpAB* vs. WT	****	<0,0001
Δ *ABpAB* vs. Δ*ABpB*	****	<0,0001

Δ *ABpAB* vs. Δ*ABpA*	****	<0,0001
WT vs. Δ*ABpB*	ns	0,0955
WT vs. Δ*ABpA*	ns	0,8941

Fig. S4 Simple linear regression	Summary	Adjusted P Value
PcoB		
ΔABpAB	ns	0,8856
*ΔABpAB*Δ(34-109)	ns	0,8142

Table S2: Strains and plasmids:

Strains	***Strains Relevant genotype or description***	***References***
ΔAB	Knockout strain for *pcoAB* genes	(17)
ΔABpA	Knockout strain for *pcoAB* genes carrying a copy of *pcoA* on the pMR10 under the control of the lac promoter; Kan^R	(17)
ΔABpB	Knockout strain for *pcoAB* genes carrying a copy of *pcoB* on the pMR10 under the control of the lac promoter; Kan^R	(17)
ΔABpAB	Knockout strain for *pcoAB* genes carrying a copy of *pcoAB* on the pMR10 under the control of the lac promoter; Kan^R,	(17)
ΔABpAB Δ(34-109)	*ΔABpAB*, the NTD of *pcoB* is deleted from aa 34 to aa 109	This study
B Δ(34-109)	The *pcoB* gene on the chromosome is deleted for the NTD from aa 34 to aa 109	
ΔABpAB Δ(34-59)	*ΔABpAB* combined with deletion of the NTD of *pcoB* from aa 34 to aa 60	This study
ΔABpAB Δ(34-85)	*ΔABpAB* combined with deletion of the NTD of *pcoB* from aa 34 to aa 85	This study
ΔABpAB N_{Ec}	*ΔABpAB* combined with replacement of the NTD of *pcoB* of *C.vibrioides* from aa 34 to aa 109 with the NTD of *pcoB* of *E.coli* from aa 25 to aa 90	This study
ΔABpAB H49-105A	*ΔABpAB* combined with point mutations of the His 49,52,67,70,71,73,74,83,105 into Ala of the NTD of *pcoB*	This study
ΔABpAB M54-102A	*ΔABpAB* combined with point mutations of the Met 54,72, 94, 102 into Ala of the NTD of *pcoB*	This study
ΔABpAB Δ(3-33)	*ΔABpAB* combined with the deletion of the NTD of *pcoB* Nterm of *pcoB* from aa 3 to aa 33	This study
ΔABpAB Δ(3-33) H49-105A	*ΔABpAB H49-105A* combined with the deletion of the NTD of *pcoB* from aa 3 to aa 33	This study

Table S3: Primers, Gbloc:

0PRIMER	***Target***	***Sequence***
**Pr1**	*pcoB*Δ(34-109) + overlap region FWD	CACGCCCATCACAGGGCCTGGATCGTCCGCGCCGACGTGG
**Pr2**	*pcoB*Δ(34-109) + overlap region RWD	GCGGACGATCCAGGCCCTGTGATGGGCGTGCGGGTCCGCC
**Pr3**	*pcoB*Δ(34-59) + overlap region FWD	GCCCATCACAGGGCCGCCCCGCAGGCCGATCCGCA
**Pr4**	*pcoB*Δ(34-59) + overlap region RWD	CTGCGGGGCGGCCCTGTGATGGGCGTGCGGGTCCG
**Pr5**	*pcoB*Δ(34-85) overlap region FWD	GCCCATCACAGGGAGCGCTTCTACAGCCCGGCCGT
**Pr6**	*pcoB*Δ(34-85) overlap region RWD	GTAGAAGCGCTCCCTGTGATGGGCGTGCGGGTCCG
**Pr7**	NEc + overlap region with Upstream NEc FWD	CACGCCCATCACAGGGGCAGCGTCTCGGCGGACCC
**Pr8**	NEc + overlap region with Downstream NEc RWD	GACGATCCAGGCCGTGATGGCGCTGTCATGGATGG
**Pr9**	Downstream NEc + overlap region with NEc FWD	CATGACAGCGCCATCACGGCCTGGATCGTCCGCGC
**Pr10**	Upstream NEc + overlap region with NEc RWD	CGCCGAGACGCTGCCCCTGTGATGGGCGTGCGGGT
**Pr11**	H49-52A point mutations+ overlap region FWD	GCGGATCCGGCTGCAGGTGCCAAGATGCCTGGACCTGAACAG
**Pr12**	H49-52A point mutations+ overlap region RWD	AGGCATCTTGGCACCTGCAGCCGGATCCGCCGGAGCGAGCAC
**Pr13**	H67-83A point mutations+ overlap region FWD	CCGATCCGGCCGCCGGCGCTGCCATGGCTGCCGCGCCGCCACCGATCCCCA CCGACGCCGCCGCCGAGCGCTTCTACAGC
**Pr14**	H67-83A point mutations+ overlap region RWD	TCGGCGGCGGCGTCGGTGGGGATCGGTGGCGGCGCGGCAGCCATGGCAGC GCCGGCGGCCGGATCGGCCTGCGGGGCGGC
**Pr15**	H105A point mutation + overlap region FWD	ATGAAGGAGGCCGGCGGCGGAACGGCCTGGATC
**Pr16**	H105A point mutation + overlap region RWD	TCCGCCGCCGGCCTCCTTCATCAGCTGGGCGCG
**Pr17**	M54-72A point mutations+ overlap region FWD	GGTCACAAGGCGCCTGGACCTGAACAGGCCGCCCCGCAGGCCGATCCGCA CGCCGGCCATCACGCGCATCACGCGCCGCC
**Pr18**	M54-72A point mutations+ overlap region RWD	CGCGTGATGCGCGTGATGGCCGGCGTGCGGATCGGCCTGCGGGGCGGCCTG TTCAGGTCCAGGCGCCTTGTGACCTGCAT

**Pr19**	M94-102A point mutations+ overlap region FWD	CCGGCCGTGGCGGCCGCGGCCCGCGCCCAGCTGGCGAAGGAGCACGGCGG
**Pr20**	M94-102A point mutations+ overlap region RWD	GTGCTCCTTCGCCAGCTGGGCGCGGGCCGCGGCCGCCACGGCCGGGCTGT
**Pr21**	*pcoB*Δ(3-33) + overlap region FWD	CGATGGAGCCGCCGCgtgATCGCTCCGGCCTCGGCGCCTC
**Pr22**	*pcoB*Δ(3-33) + overlap region RWD	CGCCGAGGCCGGAGCGATcacGCGGCGGCTCCATCGAGCG
**pcoB RWD**	Amplification RWD of *pcoB* (SacI)	cgagctcCTACCGCCAGGCGCT
**pcoA FWD**	Amplification FWD of *pcoA* (HindIII)	aagcttgtgGTTCGGGTGTTGGAAT
**Upstream pcoB FWD**	Amplification FWD 400pb upstream of *pcoB*	GCCTCTATCCGCCCAAGGACT
	Target	***Sequence***
**Gbloc**	N-ter domain sequence of pcoB from *E.coli* (270 bp)	ATGAAGCGTAATCTCAAGGCCATCCCCGTGCTCGTCGCGGGGCTCTTTACG TCCCAACTCTCCATCGCCGCCGGCAGCGTCTCGGCGGACCCGCACGCGGGG CATGATATGTCGGCGATGCAAATGCCGGCCGATGAGAATTTTACGGAGATG ACGTCGATGGAACCCATCGTGACCGAGTCCCGTACGCCCATCCCGCCCGTG ACGGACGCGGATCGCAAGGCCGCCTTCGGGAATCTCCAAGGGCACGCCAT CCATGACAGCGCCATCAAT

Table S4: Primers for RT-qPCR :

PRIMER	***Target***	***Sequence***
qRT_rpoD_F	Couple 1 forward amplification of rpoD (CCNA_03142)	GAAATGGTCGAGGCCAACCT
qRT_rpoD_R	Couple 1 reverse amplification of rpoD (CCNA_03142)	TCAGGCCGATATTGCCTTCC
PcoA_RTQ_F1	Couple 1 forward amplification of pcoA (CCNA_01015)	GCATGGGGAACATGCATCAC
PcoA_RTQ_R1	Couple 1 reverse amplification of pcoA (CCNA_01015)	CATATTGCCGGTCAGGTGGA

qPCR_pcoBF7	Amplification F of *pcoB*	cgccctattggttcgagctg
qPCR_pcoBR8	Amplification R of *pcoB*	gttgcaggatcagcctctgg
